# COVID-19: US federal accountability for entry, spread, and inequities—lessons for the future

**DOI:** 10.1007/s10654-020-00689-2

**Published:** 2020-11-02

**Authors:** William P. Hanage, Christian Testa, Jarvis T. Chen, Letitia Davis, Elise Pechter, Peg Seminario, Mauricio Santillana, Nancy Krieger

**Affiliations:** 1 Department of Epidemiology, Center for Communicable Disease Dynamics, Harvard T. H. Chan School of Public Health, Boston, MA USA; 2 Department of Social and Behavioral Sciences, Harvard T.H. Chan School of Public Health, Boston, MA USA; 3 Consultant, Boston, MA USA; 4 AFL-CIO (Retired), Washington, DC USA; 5 Computational Health Informatics Program, Boston Children’s Hospital, Boston, MA USA

**Keywords:** COVID-19, Border control, Occupational health, Health inequities, Pandemic preparedness, Trump administration

## Abstract

The United States (US) has been among those nations most severely affected by the first—and subsequent—phases of the pandemic of COVID-19, the disease caused by SARS-CoV-2. With only 4% of the worldwide population, the US has seen about 22% of COVID-19 deaths. Despite formidable advantages in resources and expertise, presently the per capita mortality rate is over 585/million, respectively 2.4 and 5 times higher compared to Canada and Germany. As we enter Fall 2020, the US is enduring ongoing outbreaks across large regions of the country. Moreover, within the US, an early and persistent feature of the pandemic has been the disproportionate impact on populations already made vulnerable by racism and dangerous jobs, inadequate wages, and unaffordable housing, and this is true for both the headline public health threat and the additional disastrous economic impacts. In this article we assess the impact of missteps by the Federal Government in three specific areas: the introduction of the virus to the US and the establishment of community transmission; the lack of national COVID-19 workplace standards and enforcement, and lack of personal protective equipment (PPE) for workplaces as represented by complaints to the Occupational Safety and Health Administration (OSHA) which we find are correlated with deaths 16 days later (ρ = 0.83); and the total excess deaths in 2020 to date already total more than 230,000, while COVID-19 mortality rates exhibit severe—and rising—inequities in race/ethnicity, including among working age adults.


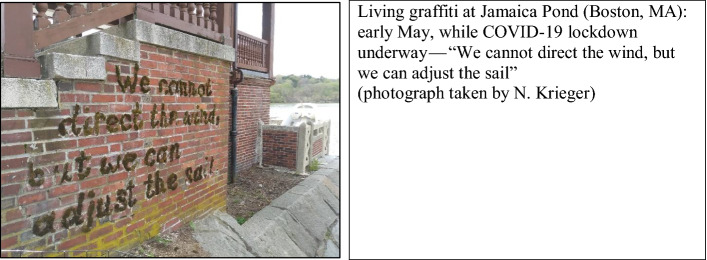
The United States (US) has been among those nations most severely affected by the first—and subsequent—phases of the pandemic of COVID-19, the disease caused by SARS-CoV-2. With only 4% of the worldwide population, the US has seen about 22% of COVID-19 deaths. Despite formidable advantages in resources and expertise, presently the per capita mortality rate is over 585/million, respectively 2.4 and 5 times higher compared to Canada and Germany [1, 2]. As we enter Fall 2020, the US is enduring ongoing outbreaks across large regions of the country. Moreover, within the US, an early and persistent feature of the pandemic has been the disproportionate impact on populations already made vulnerable by racism and dangerous jobs, inadequate wages, and unaffordable housing, and this is true for both the headline public health threat and the additional disastrous economic impacts [3–8].

The newly issued federal *Healthy People 2030* framework (released on August 18, 2020) [9], sets the nation’s objectives for the next decade, with the first two overarching goals being: (1) “Attain healthy, thriving lives and well-being free of preventable disease, injury, and premature death” and (2) “Eliminate health disparities, achieve health equity, and attain health literacy to improve the health and well-being of all.” The third is: “Create social, physical and economic environments that promote attaining the full potential for health and well-being for all” [10]. In the case of COVID-19, the US has missed the mark on all three goals.

In the following we document critical federal missteps in protecting the nation, with an eye towards guiding a better response. We start with how community transmission in the US became established in the spring. We then document complaints submitted to the US Occupational Safety and Health Administration by US workers regarding their risk of exposure to COVID-19 at work, a key site of transmission, and compare the temporal relationship of these complaints to COVID-19 cases and deaths. We conclude with an empirical analysis of excess mortality and age-specific racial/ethnic inequities in COVID-19 mortality.

## Control of borders: entry of the virus

To prevent or delay establishment of the virus, many countries have implemented symptom screening at ports of entry, sometimes together with quarantine of travelers. Such measures have enabled Singapore, Hong Kong, and Taiwan to avoid large early outbreaks, despite having been at high risk of introductions due to the volume of travelers from Wuhan (Hubei province, China), the initial epicenter of the pandemic. In the US, control of borders, including in relation to public health, rests with the federal government, an authority assumed since the founding of the country [11–15]. The administration implemented a travel ban affecting only non-US travelers from China on January 31, 2020, without any mandatory symptom screening at entry or quarantine of travelers, despite the virus at this time already known to be present in Italy, Iran, Spain, Germany, Finland, and the United Kingdom. Similarly, selective restrictions on travel from Europe were only instituted a full 6 weeks later, on March 11, 2020, at which point 830 deaths had been reported in Italy alone (by contrast, on January 31, 2020 China had reported 259 deaths). None of these restrictions applied to returning US citizens or permanent residents, despite their potential exposure to SARS-CoV-2 [16].

Hence there were ongoing opportunities for introductions of the virus to the US from Europe. Consistent with this, phylogenetic studies have found repeatedly that the great majority of SARS-CoV-2 introductions to the US were viral lineages circulating in Europe [17]. A study of the genomic epidemiology of the early pandemic in the Boston area estimated a total of 25 independent importations from Europe, 18 of which occurred prior to March 28 [18–20]. Imports included a superspreading event at a conference on February 26–27 leading to more than 90 known infections among attendees or their contacts. The index cases were two international business travelers. This introduction is estimated to be the direct ancestor of 35% of sampled genomes not associated with the conference, including samples from outbreaks in homeless shelters, so it is reasonable to suggest that this introduction alone hastened the establishment of the virus in the region and the scale of the subsequent surge of infections. As has been previously shown, by early March the most likely source of introduction to a region was from elsewhere in the United States [21]. This shows that any action to delay or prevent introductions would need to be at the national level, and while the window for effective action was brief, it was missed.

Symptom screening alone is unlikely to provide a significant barrier to a virus like SARS-CoV-2 that is capable of presymptomatic transmission [22], but may be an effective component of mitigation if supplemented by a coherent central strategy to delay case importation. While like-for-like comparisons of government responses to COVID-19 should be interpreted with caution, the benefits of such an approach are well-illustrated by Australia: like the US, Australia is a large and well-developed economy with major metropolitan cities, substantial international travel, and wide variations in population density. Also similar to the US, important elements of pandemic response were devolved to individual states, but border control is the responsibility of the central federal government. Australia had reported 300 confirmed cases on March 15 when it imposed a closure of its borders to non-Australian citizens, which was augmented by a mandatory 14-day, supervised quarantine in a hotel for all international arrivals (including Australian citizens), aggressive testing and contact tracing, and population-wide adherence to social distancing guidelines [23]. Following these local containment measures Australia saw no explosive COVID-19 case surges through the Southern hemisphere winter. Despite a recent burst of domestic transmission in the state of Victoria that resulted in the immediate re-imposition of a statewide lockdown and interstate movement restrictions [24], at present (early September 2020) Australia reports 25.6 deaths/million persons, in comparison with the US report of 585 deaths/million persons [25]. This is the difference between establishing effective national border policies and failing to do so.

## Lack of occupational health protections and community spread

The pandemic has presented different risks and rates of exposure to different sectors of US society, at different times and in different places. Health care and other essential workers who continued to work while much of the country was shut down, who work in close quarters or have contact with the public as part of their job—e.g., in transportation, critical manufacturing, food and agriculture, grocery stores, and pharmacies—have faced a greater risk of exposure and infection from COVID-19. [26–30]. Despite the designation of these workers as “essential,” an important federal failure has been the lack of standards requiring systematic collection of data on COVID-19 cases or deaths by industry and occupation. Nevertheless, information available from case reports, state health departments, media reports, and other sources show: large numbers of cases among health care workers and first responders; major outbreaks in meatpacking, and poultry plants [31]; outbreaks among prison staff (above and beyond inmates); and numerous cases in transportation, warehousing, and other essential industries, as well as in public-facing occupations (e.g., grocery stores and retail) [32]. People infected at work expose their household members—and if they are low-wage workers, they are more likely to live in crowded housing with inadequate ventilation, which increases the risk of transmission and decreases the options for people who are ill to self-isolate [7, 8, 33–35].

The federal government could have limited transmission of SARS-CoV-2 at the workplace in multiple ways. One is through its unparalleled purchasing power and ability to invoke the Defense Production Act and ensure equitable supply and distribution of PPE. However supplying PPE was delegated to a variety of actors: state and city governments, large hospital chains, and in some cases small networks of clinics [13]. Second, the federal government could have established mandatory universal paid sick leave for those unable to work due to Covid-19, but it did not. The congressional mandate for 80 h of paid COVID-related leave covered only some groups of workers. Third, the federal government could have mandated standards for occupational exposures, but it failed to act, even as some US states have done so. To date, the federal Occupational Safety and Health Administration (OSHA) has not issued any emergency or permanent standard specific to COVID-19 exposure at the workplace; additionally, as of August 13, 2020, federal OSHA, which oversees enforcement of OSHA standards in 28 states, had issued only four citations related to COVID-19 [36]. Moreover, the total number of federal OSHA inspections (of any kind) during 2020 has been reduced by two-thirds, compared to the same period in prior years [37].

Several of the 22 states with approved state OSHA plans have taken stronger regulatory and enforcement action than federal OSHA to protect workers from exposure to SARS-CoV-2. For example, Virginia issued a comprehensive emergency temporary standard on SARS-CoV-2 on July 27, 2020, and the state of Michigan has issued multiple executive orders on worker protection from COVID-19 enforced through the Michigan State OSHA plan [38, 39]. The California, Nevada, and Minnesota state OSHA plans have all issued numerous citations under other existing standards to protect workers from COVID-19 [40]. However, in most states little regulatory or enforcement action has been taken by either the federal government or the state to address workplace exposures to the virus [13, 36].

While there have been few federal OSHA citations, worker complaints to OSHA that raise concerns about workplace conditions and exposure to COVID-19 can serve to estimate hazards of exposures and risks of infection as reported by workers themselves. To our knowledge, these data have not been analyzed in relation to the population burdens of COVID-19 [41].

**Fig. 1:**
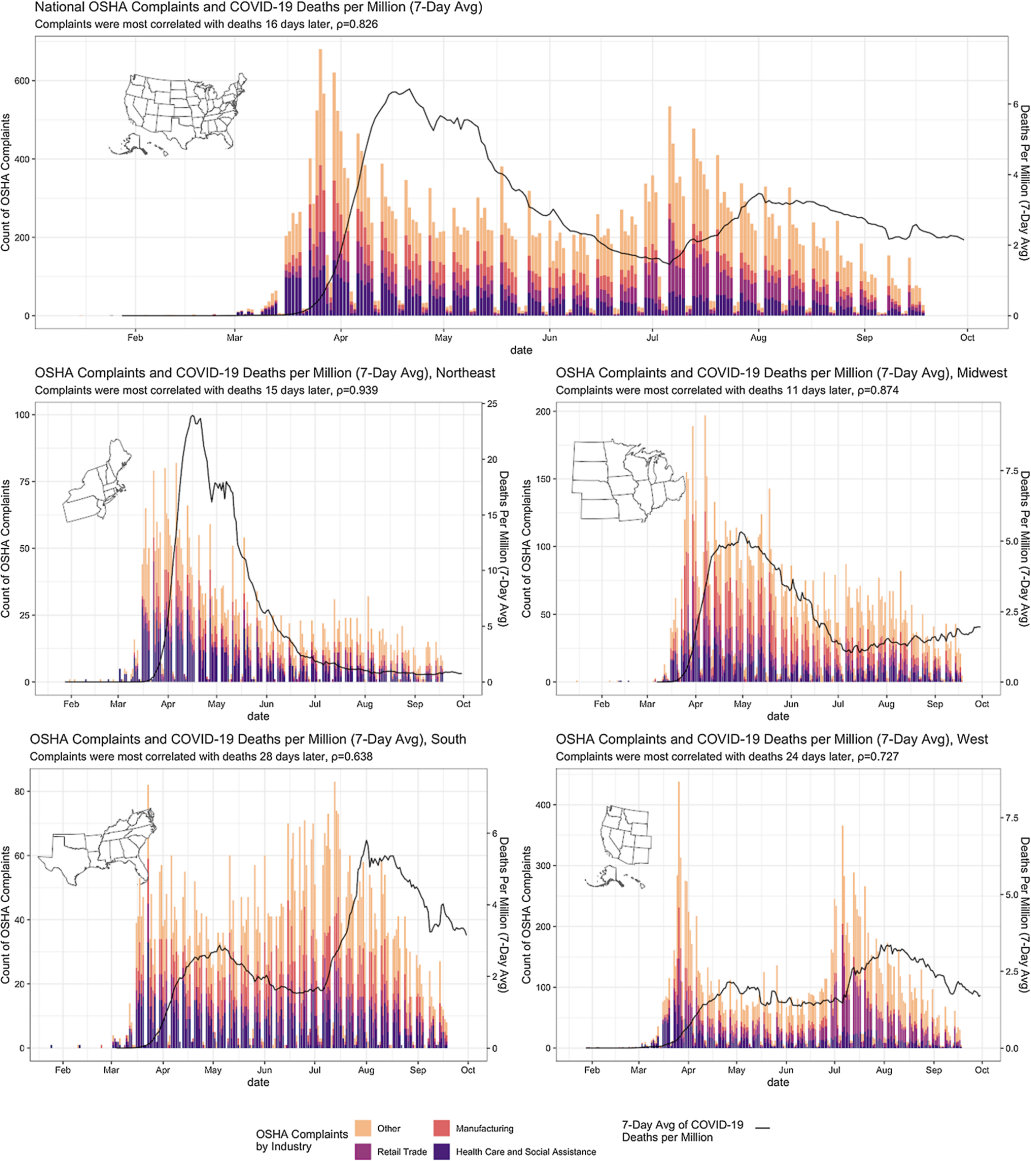
National OSHA complaints and COVID—19 deaths per million (7 Day Average), January 16-September 18, 2020

Figure 1 shows the volume of OSHA complaints (federal and state combined), broken down by four industries (manufacturing, retail, healthcare and social assistance, and other up to September 18th 2020). The total number of national complaints is shown, as are the numbers of complaints for the four main geographical regions of the country. COVID-19 mortality data from confirmed cases is also shown.

**Fig. 2:**
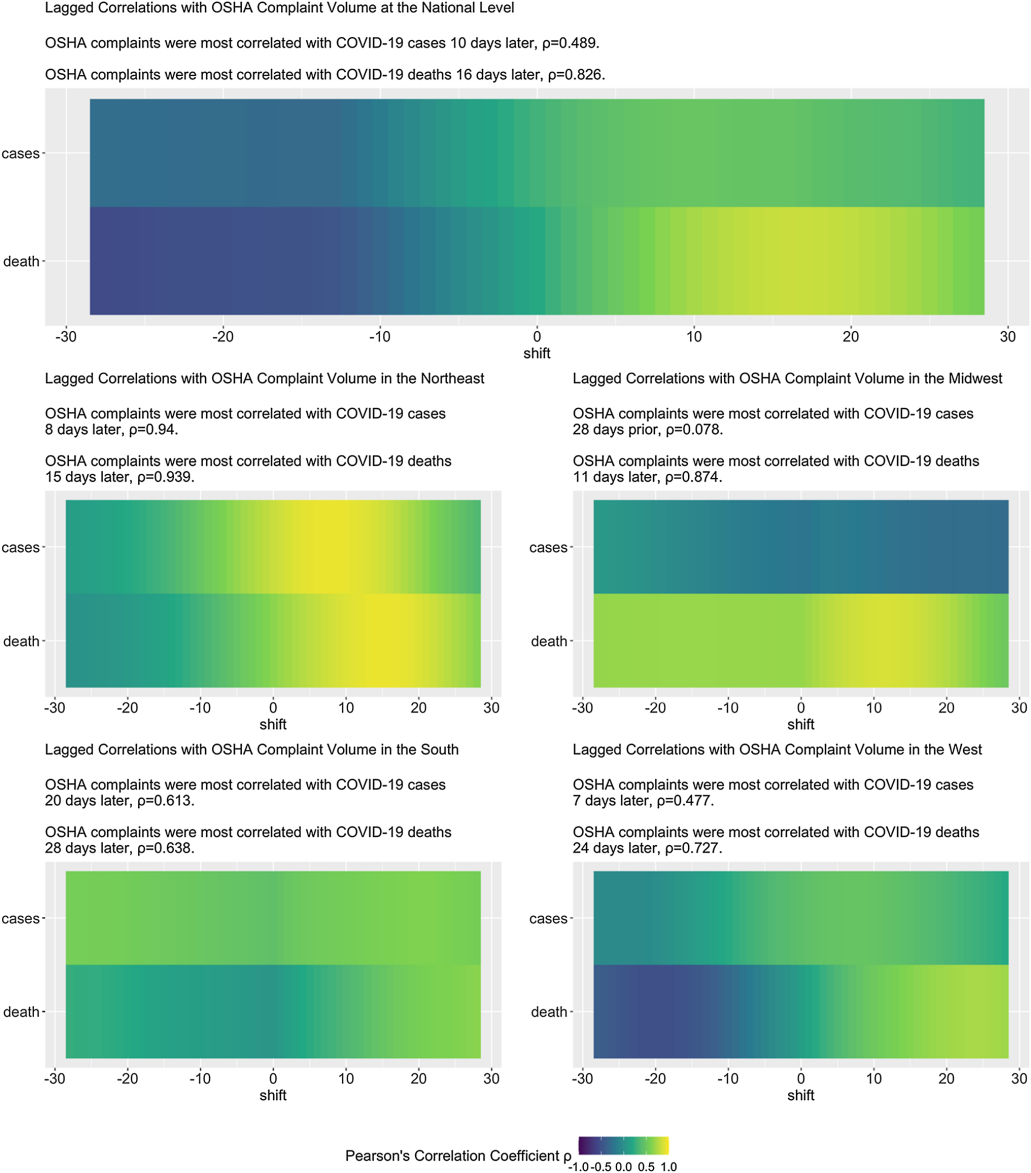
Heatmaps showing the lagged correlations between OSHA complaint volume and COVID-19 cases and COVID-19 deaths, nationally and by US region, January 16, 2020—September 30, 2020

Figure 2 in turn presents results of the time-series analysis using OSHA complaints volume, as a function of time, as a predictor and confirmed COVID-19 cases and COVID-19 deaths as the response variables. Specifically, we identified the best time lag that predicts each response variable—using OSHA complaints as a predictor—by calculating the Pearson correlation between multiple lags for each response variable and OSHA complaints.

Two findings stand out:

(1) The curve of the OSHA complaint data resembles an epidemic curve (Fig. 1), and (2) the complaints predate COVID-19 deaths (Fig. 2). At the national level, the OSHA complaints were best correlated with (a) COVID-19 deaths 16 days later ($\rho$  = 0.83), and (b) COVID-19 new confirmed cases 10 days later ($\rho$  = 0.49). Across all four subregions—Northeast, South, Midwest, West—of the country, OSHA complaints foreshadowed COVID-19 deaths by 1.5–4 weeks, with moderate to high correlations (0.64 to 0.94). Correlations with cases, however, are notably weaker in regions other than the North-East, which may be unsurprising given the volume of cases there in the spring. In general the variation in rates by geographic region and industry is substantial and mirrors the course of the pandemic—notably when considering individual industries the strongest correlation observed was between complaints in the Health Care and Social Assistance sector and deaths 20 days later ($\rho = 0.928)$. Regional correlations between complaints and reported COVID-19 cases and COVID-19 deaths (Fig. 1) suggest complaints reflect local responses to awareness of pandemic activity. These patterns point to a lack of learning from experiences across states, and the lack of enforced federal standards.

According to OSHA COVID-19 enforcement data, as of September 18, 2020, federal OSHA had opened inspections for only 191 out of 8909 worker complaints (2.1%) received related to COVID-19 [42, 43]. During this same time period, the state OSHA plans had opened 1076 inspections in response to 27,093 COVID-19 complaints received—4.0% of worker complaints for COVID-19 [42, 44].

These results further suggest that OSHA complaints have the potential to be used, in a prospective manner, to predict changes of COVID-19 activity as an additional data source in early warning systems such as those as described in [45].

We cannot determine the extent to which patterns of OSHA complaints reflect growing awareness of the pandemic and the need for adequate protection, or whether they are more direct indicators of failures that causally contributed to workplace, family, and community spread. But findings do suggest that expressed worker concerns may be an indicator of real risks, and failure to respond is a missed opportunity to intervene to mitigate disease transmission in the workplace and, in turn, the community at large. While there is not a consistent definition of “essential workers,” data suggest that people in this category disproportionately are low-income, are people of color, rely on public transportation, and live in crowded housing [7, 8, 27–29]. They also have more underlying disabilities and less access to care, which underscores the importance of mitigating exposures to SARs-CoV-2 in the workplace. Work is a key exposure risk, and to date, federal policies regarding COVID-19 and workplaces have chiefly aimed to limit the liability of employers, rather than to protect employees through, for example, mandating that employers develop an infection control plan [46]. In the following section we consider the consequences in terms of excess all-cause mortality and racial/ethnic inequities in COVID-19 mortality.

## The outcome: total deaths, inequities in premature mortality, and increasing cases

Beyond the number of confirmed COVID-19 deaths in the US (more than 215,000 as of 10/13/2020, estimates of excess deaths (comparing the same calendar period for 2020 to the average annual deaths for 2015–2019 and controlling for population growth) provide a powerful way to gauge the excess mortality due to COVID-19. Excess mortality may be due directly to the infection or be due to delays in seeking care for non-COVID illnesses or injuries; this measure also takes into account potential reductions in mortality (e.g., due to lower air pollution during the economic lockdown) as well as the seasonality of death rates [47–49].

In Table 1, we show the results of four different approaches, ranging from more to less conservative, that have been used to estimate the excess number of deaths and the excess death rate (per 100,000 person-years (p-y)), in relation to US mortality data as of September 12, 2020 [50–53]. No matter which method is used, the estimated excess number of deaths, ranging from 286,425 to 333,906, is already greater than the outer margin of what the federal government stated, on April 3, 2020, would be the upper limit of the expected death toll, i.e., 200,000 [54–56].

**Table 1:** Estimation of excess deaths during the US COVID-19 pandemic, comparing weekly 2020 death counts to the corresponding average annual 2015–2019 deaths, using 4 different methods^a^ as of September 12, 2020 (Source: Centers for Disease Control and Prevention)

Method	Crude excess	(95% CI)		Age-standardized excess rate per 100,000 person-years^b^	(95% CI)		Age-standardized cumulative incidence proportion per 100,000 population^c^	(95% CI)
1	324,813	(323,696	, 325,930)	134.5	(133.6	, 135.4)	82.5	(82.0, 83.1)
2	333,906	(332,773	, 335,038)	138.3	(137.4	, 139.2)	84.8	(84.3, 85.4)
3	286,425	(285,376	, 287,474)	118.1	(117.2	, 118.9)	72.4	(71.9, 72.9)
4	302,366	(301,289	, 303,444)	124.9	(124.0	, 125.7)	76.6	(76.1, 77.1)

^a^Method 1: sum up over weeks and then age-standardize using the direct method

Method 2: count only age strata and weeks where the excess is greater than zero, and set weeks where the excess in the age stratum is less than zero to zero

Method 3: instead of comparing to the average deaths in 2015-2019, we compare to the 95% upper bound on the average deaths for 2015-2019

Method 4: we compare to the upper bound on the average deaths for 2015-2019, but once again ignore weeks where the excess in the age stratum is less than zero

^b^Age-standardized excess rate per 100,000 person-years is computed based on dividing the age-specific excess count of deaths under the four methods by the age-specific population person-time (taking into account the age-specific 2020 population counts and the elapsed time since January 1, 2020), weighting by the year 2000 standard million, and summing over age categories

^c^Age-standardized cumulative incidence proportion per 100,000 population (i.e. a risk per capita) is computed based on dividing the age-specific excess count of deaths under the four methods by the age-specific population count in 2020, weighting by the year 2000 standard million, and summing over age categories

We additionally estimated an annual excess age-standardized death rate of 91.6 per 100,000 p-y (95% CI 89.3–93.9) based on the difference in age-standardized mortality rates for 2020 vs 2015–2019, (as opposed to scaling the estimated excess death count by the 2020 population as in Table 1). This estimate is almost double that of the third leading cause of death in the US, i.e., unintentional injuries, for which the annual age-standardized death rate was 48/100,000 p-y in 2018 (the most recent year available) [57]. The number of excess deaths in 2020 (324,813) is already well over half the number of cancer deaths for all of 2018 (609,640) [57]. It should be obvious that given that the pandemic is not over, and transmission continues in much of the country, these numbers can only increase.

Australia is again instructive, as it exhibits very little excess mortality. The most recent report from the Australian Bureau of Statistics states, “55,047 doctor certified deaths occurred between 1 January 2020 and 26 May 2020 and were registered by 30 June. This compares to a baseline average of 53,361 over the past 5 years” [58]. This translates to an excess of only 1686 deaths in Australia. In contrast, the US, with a population 13 times greater, has 154 times more excess deaths.

If the US has struggled with national pandemic response, it is instructive to compare with Europe. Like the US, European nations are advanced economies with substantial resources. Unlike the US, they are themselves independent nation states, with no overarching federal coordination, and the continent is far more densely populated than the US and has an older population. Some European nations have suffered even higher per-capita mortality from the pandemic so far than the US, among them Italy and Spain (hit hard early in the spring) and the United Kingdom. In contrast others such as Germany (like the US a federal nation with extensive porous land borders) have far less Covid-19 related mortality, and relatively little excess mortality overall including in the most at-risk age groups [59, 60]. In contrast with the US, European nations have seen few subsequent surges over the summer after initial introductions were controlled, even if cases are increasing once more in many countries. However, the overall all-cause mortality for the continent as a whole through the end of July, when outbreaks were still ongoing across the south of the US, is estimated to be 28% lower than the US [61].

We must also consider outcomes other than death. While SARS-CoV-2 is a virus that spreads via the respiratory route, it causes a systemic infection with commensurate potential for diverse long term sequelae, including important outcomes such as stroke in young, otherwise healthy adults [62], and Multisystem Inflammatory Syndrome in Children (MIS-C), a condition similar to Kawasaki syndrome that is associated with pediatric SARS-CoV-2 infection [63]. If an extremely large number of infections are permitted, even rare chronic outcomes will leave a large burden on healthcare after the pandemic. As of August 27, 2020, the number of confirmed cases in the US equaled 6.04 million—almost on par with the population of Massachusetts (6.9 million) [64]. If 0.5% (30,222) develop persistent debilitating symptoms, this would be similar to the number of new cases of cancer per year in Massachusetts (which reported an average of 31,166 per year for 2012–2016) [65]. If 5% of the 6 million cases were so afflicted, the number of persons (300,000) would exceed the 2020 national estimate for the number of US women diagnosed with breast cancer (279,100) [66]. The proportion of long-term effects of one kind or another is unknown, but it is increasingly recognized as a source of serious concern [67, 68].

**Fig. 3:**
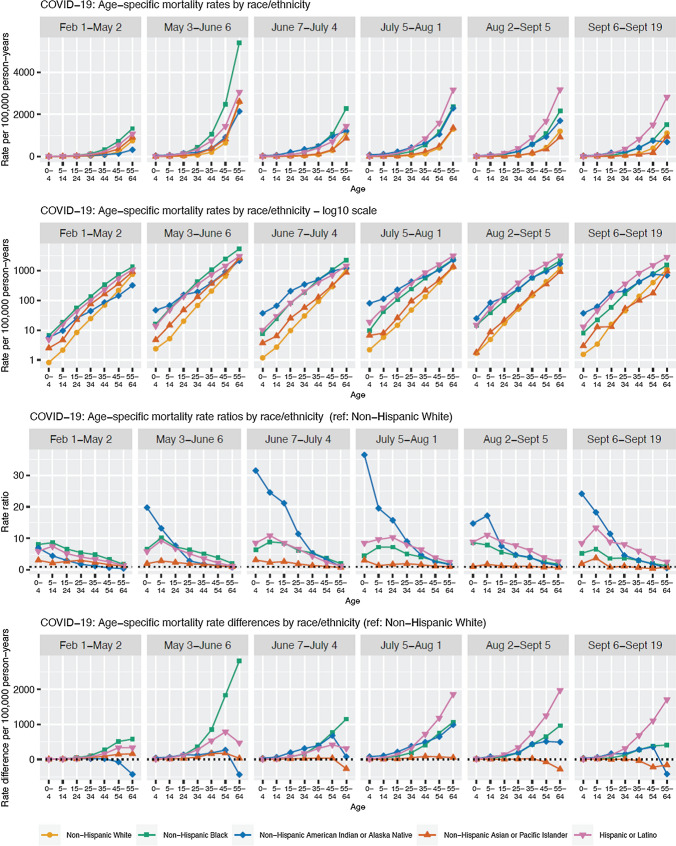
Temporal trends in US age-specific racial/ethnic COVID-19 mortality rates, rate ratios, and rate differences: February 1—September 19, 2020

Finally, despite the initial federal failure to report COVID-19 data by race/ethnicity [6], a combination of specific studies, state reporting, investigative journalism, and data trackers has revealed that a persistent feature of the pandemic has been the existence of racial/ethnic inequities in cases, hospitalizations, and mortality, especially with regard to increased risk among US Black, Latinx, and American Indian/Alaska Native populations compared to the US white non-Hispanic population [3–5, 7, 8, 69, 70]. What is less appreciated is that racial/ethnic inequities in COVID-19 mortality rates, especially among younger working-age adults, are increasing over time, especially among the Latinx and American Indian/Alaska Native populations. These trends are evident in Fig. 3, which we generated using CDC data for February 1 through September 19th, 2020 (with 99.2% of deaths assigned a race/ethnicity) [53, 71]. Specifically, the racial/ethnic inequity in age-standardized mortality rate ratios, compared to white non-Hispanics, increased over time for the five calendar periods displayed (February 1-May 2, May 3-June 6, June 7-July 4, July 5-August 1, August 2- September 5, and September 6–September 19) among working age adults (ages 25–64) who were categorized as Black non-Hispanic (p value for trend < 0.0001), Hispanic (p < 0.0001), and American Indian/Alaska Native (p < 0.0001).The estimates we report, moreover, are likely to be conservative because classification of COVID-19 deaths in part depends on testing, and inequities in access to testing, especially affecting low-income communities of color, would deflate their reported COVID-19 mortality rates [72, 73]. Moreover, the elevated mortality risk on the order of 5- to tenfold among persons under age 65 shown in Fig. 2 is unlikely to be explained by pre-existing co-morbidities, because the documented racial/ethnic mortality rate ratios for major chronic conditions (including cardiovascular disease, diabetes, and cancer) among adults under age 65 are around twofold or less [70, 74, 75].

In closing, we have documented key points at which the US federal government has failed to take the actions and collect the data needed to protect the US population from COVID-19 and the attendant toll on population health and health inequities. Despite the understandable dismay at the state of the pandemic in the United States, it is not too late to make a difference, and that difference starts with the implementation of apt policies. Those policies are too numerous to list here but have been amply documented in publications from organizations such as the Center for Global Development, which identified many of the relevant challenges early in the pandemic, stating in February: “There is an urgent but closing window to prepare for large-scale spread of the disease in the US and elsewhere. This paper recommends actions to address pressing gaps in US and global preparedness in the event that COVID-19 cannot be contained and sustained human-to-human transmission occurs beyond China” [76]. The National Association of County and City Health Officials has made the urgency of maintaining health equity in an emergency preparedness plan, as have local health officials [77, 78]. Perhaps most poignantly, a “Pandemic Playbook” produced by the National Security Council in 2016 “repeatedly (advises) officials to question the numbers on viral spread, ensure appropriate diagnostic capacity and check on the US stockpile of emergency resources” [79]. What we have seen is the consequence of not heeding that advice.

All cases and deaths cannot be prevented—that clearly is not possible with a novel pandemic—but the evidence suggests that ineffective national policies and responses [13], especially as compared to those of other wealthy nations or compared to the intricate preparation and planning by previous administrations of both parties, have been driving the terrible toll of COVID-19 and its inequities in the US. This country—and its political leaders, who bear responsibility—can and must do better.

## Data Availability

All data publicly available.
